# Suppression of genetic recombination in the pseudoautosomal region and at subtelomeres in mice with a hypomorphic *Spo11* allele

**DOI:** 10.1186/1471-2164-14-493

**Published:** 2013-07-22

**Authors:** Fatima Smagulova, Kevin Brick, Yongmei Pu, Uttara Sengupta, R Daniel Camerini-Otero, Galina V Petukhova

**Affiliations:** 1 Department of Biochemistry and Molecular Biology, Uniformed Services University of the Health Sciences, Bethesda, MD, USA; 2 Genetics and Biochemistry Branch, National Institute of Diabetes, Digestive and Kidney Diseases, NIH, Bethesda, MD, USA; 3 Present address: Inserm UMR1085-Irset 263, ave du Général Leclerc, 35042, Rennes, France; 4 Present address: Center for Investigative Proteomics, Northwick Park Institute for Medical Research, Northwick Park & St Mark’s Hospital, Harrow, Middlesex HA1 3UJ, England, UK

**Keywords:** Homologous recombination, Recombination hotspots, SPO11, Subtelomeres, Double stranded DNA breaks

## Abstract

**Background:**

Homologous recombination is the key process that generates genetic diversity and drives evolution. SPO11 protein triggers recombination by introducing DNA double stranded breaks at discreet areas of the genome called recombination hotspots. The hotspot locations are largely determined by the DNA binding specificity of the PRDM9 protein in human, mice and most other mammals. In budding yeast *Saccharomyces cerevisae*, which lacks a *Prdm9* gene, meiotic breaks are formed opportunistically in the regions of accessible chromatin, primarily at gene promoters. The genome-wide distribution of hotspots in this organism can be altered by tethering Spo11 protein to Gal4 recognition sequences in the strain expressing Spo11 attached to the DNA binding domain of the Gal4 transcription factor. To establish whether similar re-targeting of meiotic breaks can be achieved in PRDM9-containing organisms we have generated a *Gal4BD-Spo11* mouse that expresses SPO11 protein joined to the DNA binding domain of yeast Gal4.

**Results:**

We have mapped the genome-wide distribution of the recombination initiation sites in the *Gal4BD-Spo11* mice. More than two hundred of the hotspots in these mice were novel and were likely defined by Gal4BD, as the Gal4 consensus motif was clustered around the centers in these hotspots. Surprisingly, meiotic DNA breaks in the *Gal4BD-Spo11* mice were significantly depleted near the ends of chromosomes. The effect is particularly striking at the pseudoautosomal region of the X and Y chromosomes – normally the hottest region in the genome.

**Conclusions:**

Our data suggest that specific, yet-unidentified factors influence the initiation of meiotic recombination at subtelomeric chromosomal regions.

## Background

Homologous recombination is initiated by the generation of DNA double stranded breaks (DSBs) by the SPO11 protein [1]. Subsequent repair of these breaks culminates in the formation of crossing overs (COs) between homologous chromosomes that, in turn, are required for faithful chromosomal segregation [2,3]. Failure to produce at least one CO per chromosome pair leads to meiotic arrest or to the formation of aneuploid gametes [4-6]. In mammalian males, the X and Y chromosomes face a particular challenge since they share only a very short homologous area called the pseudoautosomal region (PAR). Accordingly, numerical abnormalities of sex chromosomes collectively represent the most common human aneuploidies [6].

The SPO11 protein has two major isoforms that have different expression patterns [7-11]. The beta isoform appears early and maintains a relatively constant level throughout the first meiotic prophase [11]. This form is sufficient for the production of the majority of meiotic DSBs [12]. Expression of the alpha isoform in males predominantly occurs in late prophase, beginning in early pachynema [11], and lack of SPO11α correlates with a reduction in the number of late-forming DSBs in the PAR [12]. Since mice expressing only the SPO11β isoform are also deficient in X/Y synapsis SPO11α is thought to be specifically required for efficient recombination in the PAR [12].

The majority of meiotic DSBs are formed at discreet areas of the genome called recombination hotspots [13-16]. In mice and human the hotspot locations are determined by the sequence specificity of the DNA binding domain of the PRDM9 protein [17-19]. This domain is highly polymorphic with different *Prdm9* alleles predicted to recognize dissimilar DNA sequences [18,20,21]. This leads to different hotspot locations in individuals carrying different *Prdm9* alleles (reviewed in [22]). The only hotspots that are shared between mouse strains with different *Prdm9* alleles, as well as with the *Prdm9* knockout mouse are found in the PAR and in the adjacent area [19], suggesting the existence of a *Prdm9*-independent DSB pathway. The PAR contains a large (~40 Kb) cluster of overlapping hotspots that collectively represent the hottest area of DSB formation in the mouse [23]. Such extensive DSB formation in the PAR is likely important to ensure that PAR undergoes an obligatory CO in every meiosis [24]. Although supporting evidence is not yet available, the DSB targeting to PRDM9-dependent hotspots could be explained, at least in principle, by a physical interaction between the DSB machinery and the PRDM9 protein (see [19] for discussion). However, the mechanisms that target DSBs to the PAR are not understood.

In this study we generate a *Gal4BD-Spo11* mouse carrying a hypomorphic *Spo11* allele that is deficient in the formation of DSBs in the PAR. This deficiency does not represent a specific defect in the PRDM9-independent DSB pathway, because PRDM9-dependent hotspots are also depleted in the region adjacent to the PAR in these mice. Furthermore, DSB reduction is also apparent at the subtelomeric regions of other chromosomes as well. Our data suggest that specific factors influence early steps of homologous recombination in subtelomeric regions including the PAR, and that the *Gal4BD-Spo11* mice have a specific defect that compromises the proper execution of the recombination program in these areas.

## Results and discussion

### Generation of the *Gal4BD-Spo11* knock-in mouse

**Figure 1:**
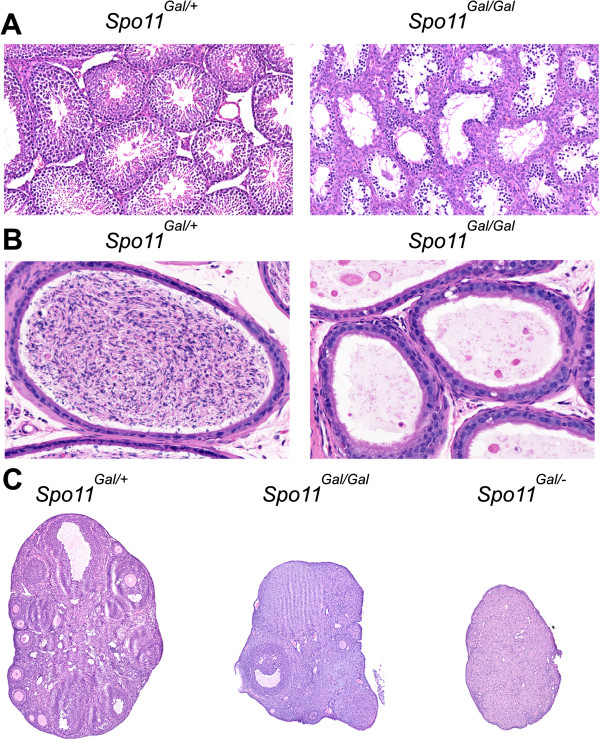
**The *Gal4BD-Spo11 *allele is insufficient to maintain normal fertility.** H&E-stained histological sections are shown. “Gal” indicates the *Gal4BD-Spo11* allele, “+” indicates the wild type allele and “–”indicates the null allele of the *Spo11* gene. **A**. Testes. Note the abundance of spermatocytes, but lack of spermatids in the seminiferous tubules of *Spo11*^*Gal/Gal*^ mice. **B**. Epididymus. Note the lack of spermatozoa in the *Spo11*^*Gal/Gal*^ mice. **C**. Ovaries. A few follicles are evident in the *Spo11*^*Gal/Gal*^ 3 month old females, but none in the *Spo11*^*Gal/-*^ mice.

The yeast Gal4 transcription factor binds to the promoters of several *S. cerevisiae GAL* genes through its N-terminal DNA-binding domain (reviewed in [25]). This domain recognizes the CGGN_11_CCG consensus sequence and, when attached to the yeast Spo11 protein, it is able to tether Spo11 to Gal4 recognition sites leading to the formation of Gal4BD-Spo11 specific recombination hotspots [26-28]. To evaluate if such tethering is possible in mice we used gene targeting in embryonic stem cells to introduce the DNA fragment coding for the DNA binding domain of the yeast Gal4 protein upstream of the start codon of the mouse *Spo11* gene (Additional file 1: Figure S1). The expression and correct splicing of the resulting gene was confirmed by sequencing PCR fragments generated from cDNA of the *Gal4BD-Spo11* homozygous (*Spo11*^*Gal/Gal*^) mice. We confirmed that both major isoforms of SPO11 – alpha and beta – were transcribed (data not shown). Nevertheless, testes of the *Spo11*^*Gal/Gal*^ mice were reduced in size and germ cells beyond the spermatocyte stage were absent (Figure 1A). Furthermore, no sperm was detected in the epididymus (Figure 1B) indicating that *Spo11*^*Gal/Gal*^ males were infertile. Similarly, *Spo11*^*Gal/Gal*^ ovaries were smaller than in wild type and showed a greatly diminished number of follicles (Figure 1C). Young *Spo11*^*Gal/Gal*^ females did produce progeny, however no viable litters have been obtained from mice older than 6 months, suggesting a premature cessation of fertility. Females carrying only one copy of the *Gal* allele and one *null* copy of *Spo11* (*Spo11*^*Gal/–*^) showed a further reduction in ovarian size, no visible follicles and an underdeveloped uterus, indicating that a single *Gal4BD-Spo11* allele is insufficient to maintain fertility (Figure 1C and data not shown).

### The number of meiotic DSBs is reduced in the *Spo11*^*Gal/Gal*^ mice

**Figure 2:**
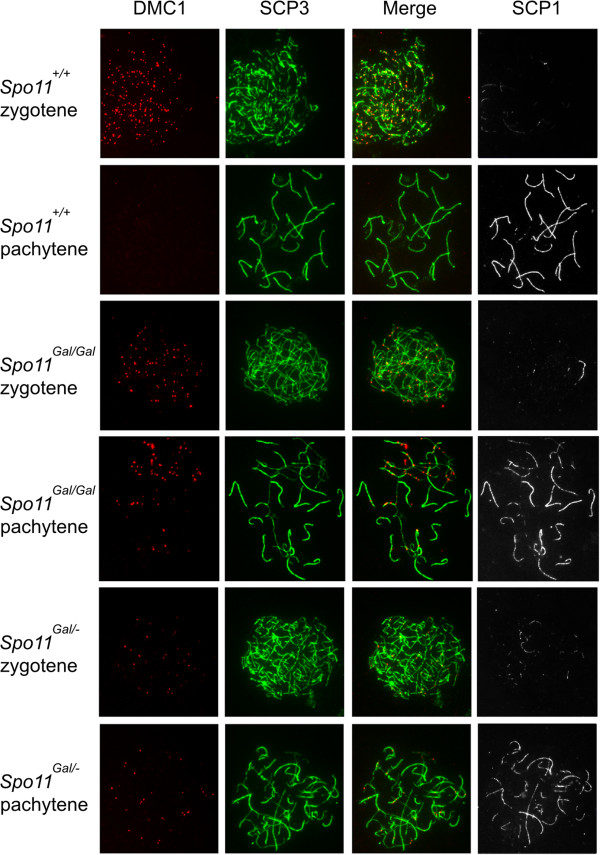
**The number of meiotic DSBs is reduced in *Gal4BD-Spo11 *males.** Chromosomal spreads from earlier (zygotene) and later (pachytene) stage spermatocytes were immunostained with antibodies to the DMC1 protein (red) to indicate the appearance and repair of DSBs in wild type mice and mice carrying one or two *Gal4BD-Spo11* alleles. Chromosome cores were stained with antibodies to the axial element component SCP3 (green), and progression of homologous synapsis was monitored with antibodies to the component of the central element of the synaptonemal complex SCP1 (white).

**Figure 3:**
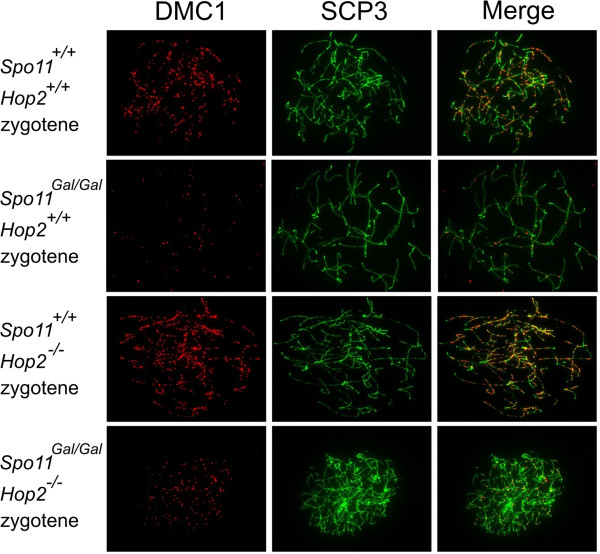
**The number of meiotic DSBs is reduced in *Gal4BD-Spo11 *females.** Chromosomal spreads from E15.5 ovaries were immunostained with antibodies to the DMC1 (red) and SCP3 (green) proteins.

**Figure 4:**
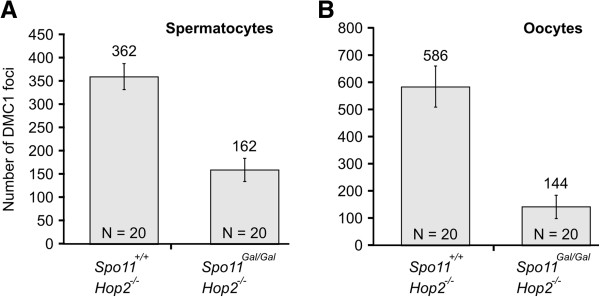
**Estimate of the number of meiotic DSBs in *Gal4BD-Spo11 *mice. A**. The number of DMC1 foci in spermatocytes. **B**. The number of DMC1 foci in oocytes. The number of analyzed cells is indicated within the bars. The foci were counted at late zygotene stage.

The yeast counterpart of the chimeric *GAL4BD-SPO11* gene complements the *SPO11*Δ mutation, indicating that the fusion Gal4BD-Spo11 protein is proficient at DSB formation [26]. To determine whether DSBs are formed in the *Spo11*^*Gal/Gal*^ mice we immunostained chromosomal spreads prepared from spermatocytes (Figure 2) or oocytes (Figure 3) with antibodies to the DMC1 protein, known to bind to the single stranded DNA tails of DSBs [29,30]. Although DMC1 foci were observed, their number was significantly reduced compared to wild type. Precise counting of DSBs is complicated by the dynamic nature of DSB formation and repair. While one can estimate the steady state level of DMC1 foci on each chromosomal spread, the DSBs that have already been repaired and the DSBs that have not yet formed will be under-counted. This problem is particularly pronounced when mutants with delayed or defective DSB repair are compared to wild type. To eliminate the effect of possible timing differences in the formation and/or repair of DSBs between *Spo11*^*Gal/Gal*^ and *Spo11*^*+/+*^ mice, we compared the number of DMC1 foci on a *Hop2*^*−/−*^ genetic background, i.e., in *Spo11*^*Gal/Gal*^*Hop2*^*−/−*^*versus Spo11*^*+/+*^*Hop2*^*−/−*^ cells. The HOP2 protein is directly involved in the repair of meiotic DSBs [31-33], accordingly, *Hop2*^*−/−*^ mice undergo meiotic arrest at the stage when all DSBs are already produced but none are repaired [34]. Therefore, the analysis of the mutants on the *Hop2*^*−/−*^ background will reduce counting bias resulting from DSB formation/repair dynamics. We found that the number of DMC1 foci in *Spo11*^*Gal/Gal*^*Hop2*^*−/−*^ spermatocytes was approximately 2 fold lower than that in the *Spo11*^*+/+*^*Hop2*^*−/−*^ spermatocytes (Figure 4). Moreover, in *Spo11*^*Gal/–*^ spermatocytes, where one *Gal* and one null allele of *Spo11* were present, the number of DMC1 foci was reduced even further – to approximately 1/6^th^ of the wild type level (data not shown). The reduction of the DSB number in *Spo11*^*Gal/Gal*^*Hop2*^*−/−*^ females was more profound than in males, with only 1/4^th^ of the normal number of DMC1 foci being formed (Figure 4).

**Figure 5:**
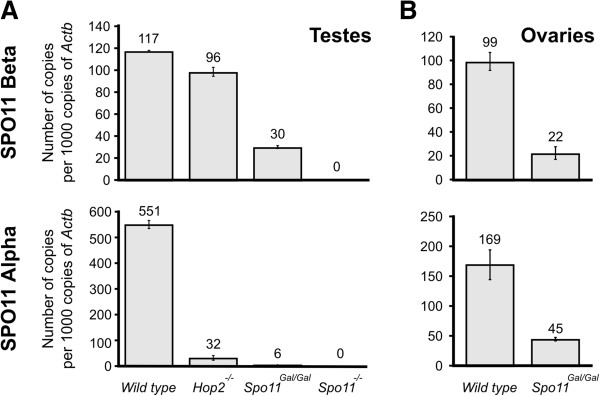
**The level of Gal4BD-SPO11 mRNA is reduced compared to wild-type SPO11 mRNA levels.** QPCR analysis of SPO11-β (top) and SPO11-α (bottom) mRNA levels in **(A)** testes and **(B)** ovaries. Note that in males the alpha isoform of SPO11 is expressed later than the beta isoform [11], resulting in drastic reduction of the alpha isoform mRNA levels in arrested *Hop2*^*−/−*^ and *Gal4BD-Spo11* spermatocytes.

To understand what could be the reason for the reduced number of DSBs we examined the mRNA levels of the chimeric GAL4BD-SPO11 protein in testes and ovaries by quantitative PCR. *Gal4BD-Spo11* male mice undergo meiotic arrest (Figure 1A and see below) and do not produce spermatids, which normally account for a large fraction of the testis germ cell population. Therefore, the cell type composition in testes of the *Gal4BD-Spo11* and wild type mice is vastly different, creating a challenge for a meaningful comparison of gene expression levels. To minimize this difference we again employed *Hop2*^*−/−*^ mice, which undergo meiotic arrest at a similar stage [34] as the *Gal4BD-Spo11* mice. We found that the mRNA level of the GAL4BD-SPO11 protein in the *Gal4BD-Spo11* mice is significantly lower than the mRNA level of the SPO11 protein in wild type or in *Hop2*^*−/−*^ mice (Figure 5). More specifically, in *Gal4BD-Spo11* males, the level of the beta isoform was reduced 3.3 fold compared to that in *Hop2*^*−/−*^ mice, and the level of the alpha isoform was reduced 5.3-fold (Figure 5A). In *Gal4BD-Spo11* females the expression of the beta isoform was reduced 4.5 fold compared to wild type mice and the expression of the alpha isoform was reduced 3.8 fold (Figure 5B). These data indicate that the mRNA level of the chimeric GAL4BD-SPO11 protein is profoundly reduced and that the observed reduction in the number of introduced DSBs could be, at least in part, a consequence of this reduction. It is also conceivable that the addition of the Gal4 binding domain to the mouse SPO11 protein might compromise the activity of SPO11, contributing to the reduced DSB count in *Gal4BD-Spo11* mice.

### Homologous chromosome synapsis in *Spo*^*Gal/Gal*^ mice is incomplete

**Figure 6:**
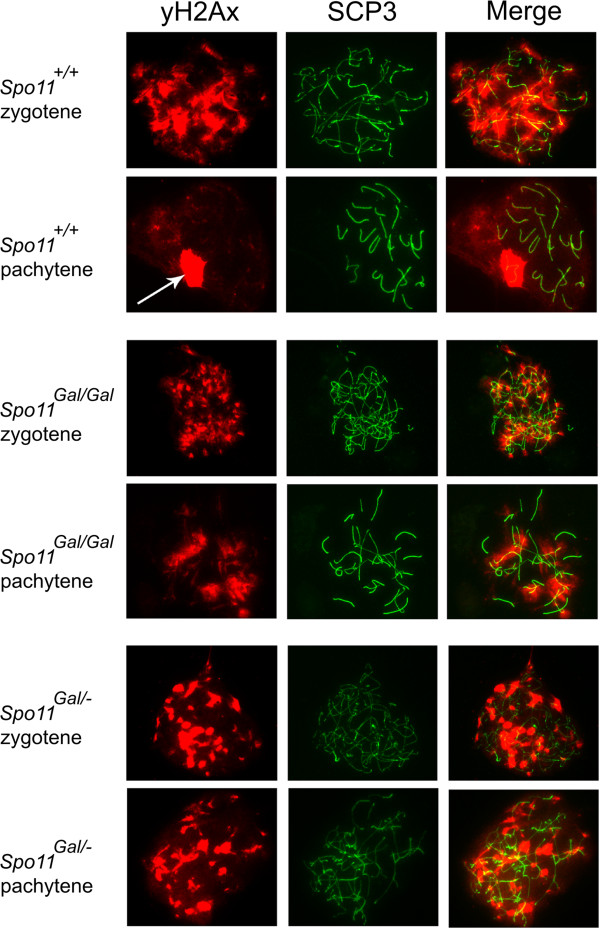
**DSB repair in *Gal4BD-Spo11 *spermatocytes is incomplete.** Chromosomal spreads from earlier (zygotene) and later (pachytene) stage spermatocytes were immunostained with antibodies to γH2AX (red) and SCP3 (green). Note the disappearance of γH2AX staining at homologously synapsed autosomes in later stage wild type and *Spo11*^*Gal/Gal*^ spermatocytes consistent with proper repair of DSBs. γH2AX persist in *Spo11*^*Gal/-*^ spermatocytes and on asynapsed chromosomes in *Spo11*^*Gal/Gal*^ spermatocytes. Sex body (white arrow) is evident in wild type, but not *Gal4BD-Spo11* spermatocytes.

**Figure 7:**
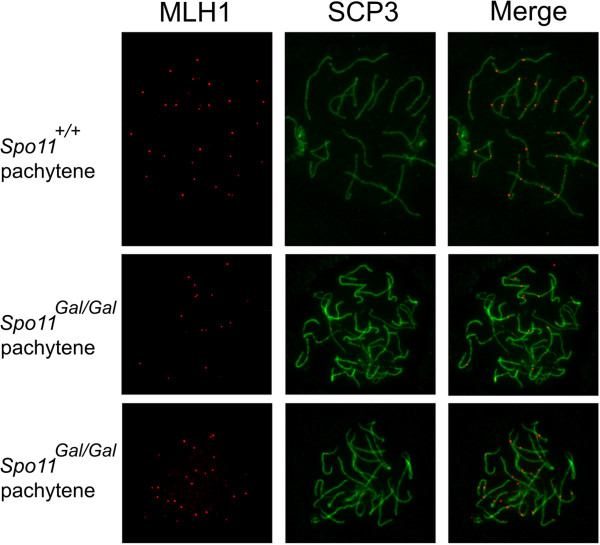
**MLH1 foci are formed in a fraction of *Spo11***^***Gal/Gal***^**oocytes.** Chromosomal spreads from E18.5 ovaries were immunostained with antibodies to MLH1 (red) and SCP3 (green). Note incomplete synapsis and consequent reduction in the number of MLH1 foci in *Spo11*^*Gal/Gal*^ oocytes.

The formation of meiotic DSBs is followed by rapid phosphorylation of histone H2AX [35]. During break repair homologous chromosomes are brought together and align throughout their entire length within a proteinaceous structure called the synaptonemal complex (SC) (reviewed by [36]). This coincides with the disappearance of the phosphorylated H2AX (γH2AX) and subsequent localization of the MLH1 protein to the sites of crossovers [37]. We analyzed homologous synapsis in *Gal4BD-Spo11* spermatocytes by immunostaining the SCP1 protein, a component of the central element of the SC [38] (Figure 2). Although a number of chromosomes in *Spo11*^*Gal/Gal*^ spermatocytes appeared properly synapsed, no cells had undergone complete synapsis. A large fraction of chromosomes synapsed only partially or formed branched structures indicative of non-homologous synapsis. γH2AX staining was lost from those chromosomes that had undergone complete synapsis, indicating successful DSB repair (Figure 6). Partially synapsed and branched chromosomes retained substantial γH2AX staining. Synaptic defects were even more prominent in the *Spo11*^*Gal/–*^ spermatocytes, indicating that a further reduction in the number of DSBs further compromises homology search and homologous synapsis (Figures 2 and 6). Although some oocytes completed meiosis and resulted in a progeny, the majority showed defects in homologous synapsis. Nevertheless, unlike in spermatocytes (data not shown), MLH1 foci were evident in oocytes even when only a fraction of homologous chromosomes were successfully synapsed (Figure 7).

The X and Y chromosomes synapse and recombine at the short region of homology within the PAR [24]. Unsynapsed parts of X and Y trigger transcriptional silencing of sex chromosomes associated with their assembly into a specialized chromatin domain called the sex or XY body (reviewed by [39-41]). At this stage the X/Y chromatin is decorated by a number of proteins including γH2AX. We found that a sex body was not assembled in the *Spo11*^*Gal/Gal*^ spermatocytes, as no area of dense and discrete γH2AX staining was observed (Figure 6). The failure of sex body formation and impairment of transcriptional silencing is common in recombination mutants with compromised DSB repair and homologous synapsis (reviewed by [42]). Transcriptional silencing of sex chromosomes is required for meiotic progression beyond the pachytene stage in males [42,43], potentially explaining the dimorphic phenotype (sterility of males and fertility or subfertility of females) in a number of mouse mutants with mild to moderate synaptic defects; e.g., *Sycp3*^*−/−*^[44,45], *H2AX*^*−/−*^[46], *Sycp2*^*−/−*^[47], *Dmc1*^*Mei11/+*^[48], *Brca1*^*Δ11/Δ11*^*p53*^*+/−*^[49], *Spo11β-only*[12]. Similar mechanisms are likely to be the reason for the dimorphic phenotype observed in *Gal4BD-Spo11* mice.

### The mouse GAL4BD-SPO11 fusion protein can target meiotic DSBs to Gal4 consensus binding sites

We have mapped the distribution of DSBs in the *Gal4BD-Spo11* mouse using anti-DMC1 chromatin immunoprecipitation (ChIP) followed by high throughput sequencing [19,50]. To increase the sensitivity of the hotspot detection we have also mapped DSBs in the *Gal4BD-Spo11* mice on the *Hop2* knockout background (*Spo*^*Gal/Gal*^*Hop2*^*−/−*^). This allowed identification of 13,445 hotspots. 98% of the hotspots detected in *Spo11*^*Gal/Gal*^ were also present in *Spo11*^*Gal/Gal*^*Hop2*^*−/−*^ mice, reaffirming that the *Hop2* mutation does not affect the distribution of meiotic DSBs genome-wide [23]. We have compared the distribution of *Gal4BD-Spo11* hotspots to hotspots in wild type mice [19] and to those in *Hop2* knockout mice. We found that 97.7% of the top 10,000 *Gal4BD-Spo11* hotspots correspond to wild type hotspots. When all 13,445 *Gal4BD-Spo11* hotspots are considered the overlap between *Gal4BD-Spo11* and wild type hotspots is 94.7%. Therefore, the overall distribution of recombination hotspots in *Gal4BD-Spo11* mice is not affected. The relative strength of the hotspots shared between wild type and *Gal4BD-Spo11* mice is also highly correlated (Additional file 2: Figure S2).

**Figure 8:**
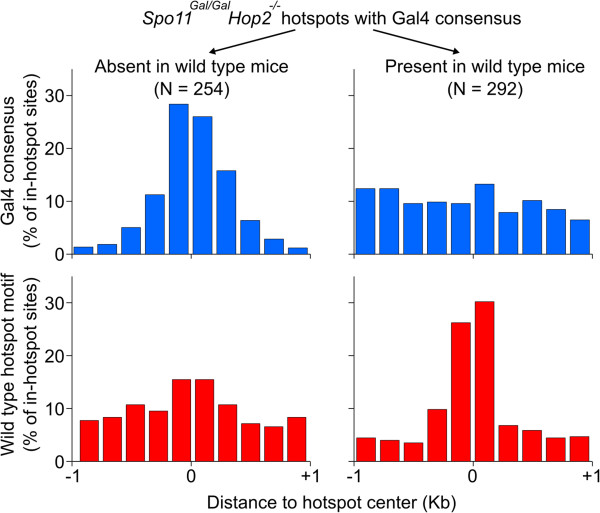
**A subset of *Gal4BD-Spo11 *hotspots is targeted to the Gal4 consensus.***Gal4BD-Spo11* hotspots containing a match to the Gal4 DNA binding consensus were sub-divided into those found in the wild type strain (C57Bl/6) and those absent in the wild type. The coverage of the Gal4 consensus and of the previously identified C57Bl/6 hotspot consensus motif were calculated around the hotspot centers.

There are 81,710 Gal4 consensus CGGN_11_CCG sequences present in the mouse genome. Approximately 2% of DSB hotspots overlapped these sites in either wild type or *Hop2*^*−/−*^ mice (Table 1). However, when the *Spo11*^*Gal/Gal*^*Hop2*^*−/−*^ mice were examined, the number of consensus-bearing hotspots increased to ~4%. These extra hotspots are weak, and although some of them are also apparent in the *Spo11*^*Gal/Gal*^ mice upon visual examination, they were beyond the detection threshold in this sample (Table 1). Out of 546 *Gal4BD-Spo11* hotspots with a Gal4 consensus site 292 were also present in wild type mice (Figure 8). A prominent peak in the distribution of wild type hotspot motif [19] was found at the center of these hotspots, whereas the distribution of the Gal4 consensus appeared random. The remaining 254 *Gal4BD-Spo11* hotspots with a Gal4 consensus were *Gal4BD-Spo11*-specific. We found that the Gal4 recognition sequence rather than the hotspot consensus motif is enriched at the centers of these hotspots. Furthermore, only 3% of these *Gal4BD-Spo11*-specific hotspots overlapped PRDM9-dependent histone H3 lysine 4 trimethylation marks (H3K4me3). In aggregate, these data indicate that a small fraction of DSBs in the *Gal4BD-Spo11* mice are likely targeted through a PRDM9-independent mechanism by tethering of the GAL4BD-SPO11 protein to Gal4 binding sites. In addition to the H3K4me3 introduced by the PRDM9 protein, gene promoters and enhancers are also decorated with H3K4me3 [51-53]. We have previously found that such sites represent preferred DSB formation loci in mice lacking *Prdm9*[19]. We now show that 84% of Gal4BD-Spo11-specific hotspots overlap transcription start sites. Importantly, the vast majority (96%) of *Gal4BD-Spo11*-specific hotspots overlap H3K4me3, indicating that H3K4me3 marks or/and events preceding H3K4 trimethylation are still essential when DSB targeting is GAL4-mediated.

**Table 1:** Gal4BD-SPO11 directs a population of hotspots to the sites with Gal4 consensus motif

**Sample**	**Sequenced ssDNA tags, ×10**^**6**^	**Identified hotspots**	**Hotspots with Gal4 consensus**		**Hotspots present in the wild type mice**	
Wild type	49.4	18,313	416	(2.3%)	18,313	(100%)
*Hop2*^*−/−*^	3.4	8,221	204	(2.5%)	8,036	(97.7%)
*Spo11*^*Gal/Gal*^	17.5	10,313	224	(2.2%)	10,086	(97.8%)
*Spo11*^*Gal/Gal*^*Hop2*^*−/−*^	46.3	13,445	546	(4.1%)	12,730	(94.7%)

The genotype of mouse strains is listed in the left column. Sequenced ssDNA tags indicate the number of ssDNA-derived fragments (e.g., those derived from ssDNA tails of DSBs) that have been sequenced for this analysis. The number of hotspots identified in each mouse strain is also indicated (column 3) and the overlap of these hotspots with the hotspots found in the wild type mice (“wild type” sample) is specified (column 5). The number of hotspots that contain Gal4 consensus motif (column 4, left) and the percentage of Gal4-containing hotspots in the total number of hotspots identified in the corresponding samples (column 4, right) are shown to demonstrate the increase in the Gal4-containing hotspots in the *Spo11*^*Gal/Gal*^*Hop2*^*−/−*^ mice (see text for more explanation).

### *Gal4BD-Spo11* mice lack a DSB hotspot cluster in the PAR

**Figure 9:**
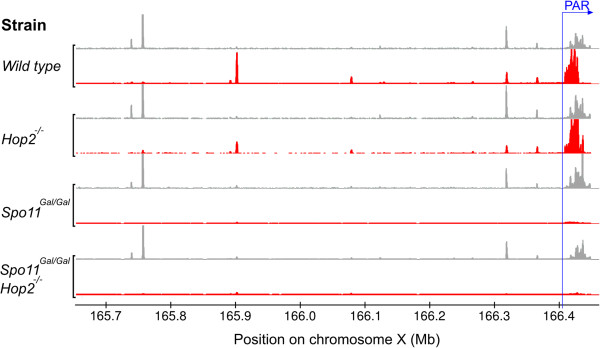
***Gal4BD-Spo11 *mice lack DSB hotspot cluster in the PAR.** Red tracks represent the DSB coverage (ssDNA fragments). Grey tracks represent the H3K4me3 coverage (tags).

We have previously demonstrated that the PAR carries a large cluster of overlapping DSB hotspots [23] that are most likely required to ensure an obligate DSB (and CO) in the PAR. Although individual hotspots cannot be resolved within the hotspot cluster it is clear that a large fraction, if not all, PAR hotspots are fundamentally different from those in the rest of the genome, because their formation does not depend on PRDM9 [19]. The formation of DSBs in the PAR has been reported to occur in two rounds: one (early) set is introduced at the same time as the majority of the autosomal breaks and the second one is introduced at a relatively late stage, presumably, by the alpha isoform of the SPO11 protein [12]. We found that *Gal4BD-Spo11* mice are severely deficient in DSB formation in the PAR (Figure 9). The contribution of the DSBs in the PAR hotspot cluster to the total number of DSBs introduced genome-wide in *Hop2*^*−/−*^ mice is estimated as 0.33%, but drops over 30-fold (to 0.01%) in the *Spo11*^*Gal/Gal*^*Hop2*^*−/−*^ (data not shown). Although this defect may be explained to some extent by the extremely low expression of the SPO11 alpha isoform in the mutant, the complete lack of SPO11 alpha has been reported to result in only a 3-fold reduction of the DSBs in the PAR [12]. Reduced expression of the beta isoform in the *Gal4BD-Spo11* mice could be another reason for the DSB reduction. However, the overall number of DSBs in the *GAL4BD-Spo11* mice (presumably, introduced by the beta isoform) is reduced only 2.2-fold. It is therefore likely that, in addition to reduced protein level, a specific defect of the GAL4BD-SPO11 protein contributes to the drastic reduction of the DSBs in the PAR.

### *Gal4BD-Spo11* mice exhibit a deficiency of DSBs at chromosomal ends

**Figure 10:**
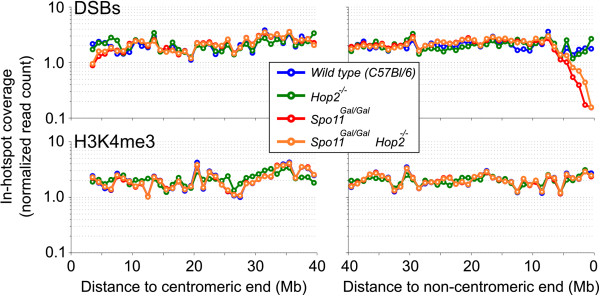
**DSBs are depleted in the autosomal subtelomeric regions of *Gal4BD-Spo11 *mice.** The coverage of DSBs (top panels) and H3K4me3 (bottom panels) in the regions corresponding to the wild type hotspots was calculated in 1 Mb windows across all autosomes. Values were normalized as a percentage of total in-hotspot coverage. DSB depletion is evident at the non-centromeric (right) chromosomal ends in *Gal4BD-Spo11* mice. PRDM9-dependent H3K4me3 coverage in these regions does not change from the chromosomal average. Note that the sequence of the first 3 Mb of centromeric ends (left) of mouse chromosomes is not available.

The PAR is located at the end of the X and Y chromosomes. We therefore examined whether DSB formation in *Gal4BD-Spo11* mice is also affected at the ends of autosomes. Indeed, we found that the strength of DSB hotspots near chromosome ends is significantly reduced in *Spo11*^*Gal/Gal*^ and *Spo11*^*Gal/Gal*^*Hop2*^*−/−*^mice (Figure 10), and as a consequence, many subtelomeric hotspots that are present in wild-type mice are not detected. This phenomenon was not observed in *Hop2* knockout mice indicating that the effect is not related to the meiotic arrest in *Gal4BD-Spo11* spermatocytes.

We propose that specific suppression of homologous recombination at subtelomeres of the *Gal4BD-Spo11* mice indicates the potential difference in the DSBs formed in these areas compared to DSBs in the rest of the genome. For example, it is conceivable that DSB formation close to chromosome ends is influenced by telomere attachment to the nuclear periphery [54] or by a specific chromosome organization and chromatin structure in the telomere-adjacent regions. Specific interactions may occur in these areas between the DSB machinery and proteins enriched at telomeres and/or subtelomeres. In fact, one such protein has recently been implicated in the regulation of transcription [55]. Incidentally, SPO11 itself was reported to bind to the telomeres of mouse embryonic stem cells [56]. It is therefore possible that the GAL4BD-SPO11 protein alone or as a component of the DSB formation complex is deficient in overcoming the inhibitory effects of the specific chromatin environment of subtelomeric regions. Such a deficiency can result from (i) the inability of GAL4BD-SPO11 to interact with a particular protein or protein complex, (ii) attenuated GAL4BD-SPO11 DNA binding in the context of such a chromatin environment or even (iii) a defect in GAL4BD-SPO11 removal from DNA in these regions that would prevent processing of meiotic DSB ends and loading RAD51 and DMC1. It is important to note that PRDM9-dependent H3K4me3 is introduced normally in hotspot-depleted regions of *Gal4BD-Spo11* subtelomeres (Figure 10), therefore PRDM9 accessibility to these areas is not an issue.

## Conclusions

Although the pivotal role of PRDM9 in defining the positions of individual recombination hotspots is established, a large number of potential PRDM9 binding sites in the genome are not being utilized. It is not clear what makes such sites refractory to the DSB machinery, but higher order chromatin structure and chromosome organization within the nucleus are likely to play a role. These features might provide slowly evolving physical constraints, which, according to a two-stage model of recombination initiation [57], ensure relatively similar recombination frequencies over large chromosomal domains between individuals in spite of *Prdm9*-dependent variability at individual hotspots within such domains. The *Gal4BD-Spo11* mutant shows a domain-specific defect in initiation of homologous recombination without visible changes in the rest of the genome. This model will be instrumental in dissecting specific interactions occurring between subtelomeric chromatin and the DSB machinery and in providing molecular insights into the megabase-scale control of initiation of homologous recombination.

## Methods

### Mouse strains

All animal procedures have been approved by the USUHS Animal Care and Use Committee or were performed according to NIH Guide for the Care and Use of Laboratory Animals.

*Gal4BD-Spo11* mice were generated on a C57Bl/6 background. The targeting construct was based on the pLoxpNEO vector [58] (a gift from Dr. Chu-Xia Deng, NIH). The *Spo11* gene was obtained from the BAC clone RP23-52C7 (CHORI). A 4.3 Kb HincII/SmaI fragment of the 5′ region of the mouse *Spo11* gene including the first exon was subcloned, and the DNA corresponding to the Gal DNA binding domain (from pGBKT7 plasmid, Clontech) was inserted upstream of the start codon of the *Spo11* gene. The resulting fragment was then cloned into the pLoxpNEO vector upstream of the *NEO* gene cassette (Additional file 1: Figure S1A). A 3.8 Kb XmaI/XmaI fragment containing exon II of the *Spo11* gene was cloned between the *NEO* and *TK* genes. Gene targeting and blastocyst injections were performed by Xenogen Biosciences Corporation (Caliper Life Sciences). Confirmation of targeting was done by Southern blot analysis (Additional file 1: Figure S1B and S1C). To excise the NEO targeting cassette *Gal4BD-Spo11* heterozygous mice were crossed with a CRE-expressing mice (Jackson Laboratories stock # 003724) and the excision was confirmed by PCR. Expression of the correctly spliced Gal4BD-Spo11α and Gal4BD-Spo11β isoforms have been confirmed by RT PCR followed by sequencing. *Gal4BD-Spo11* mice were genotyped by PCR using the following primers: Gal/dir: CTCAGAGCGGCTCCGCATCC; Gal/rev: GGCGCCACGAGGAACCTTCC. *Spo11*^*−/−*^ mice (strain *deltaSpo.BC/B6*) have been produced by the targeted deletion of exons 2–6 of the *Spo11* gene resulting in the absence of the SPO11 protein (Romanienko and Camerini-Otero, unpublished). The phenotype of this knockout strain is identical to the phenotype of the previously described *Spo11*^*−/−*^ strain *Spo11*^*tm1Rdco*^[8]. *Hop2* knockout mice have been previously described [34]. C57Bl/6 was used as a wild type strain and all mutant strains were on a C57Bl/6 background. Adult (2–6 month old) mice were used for the analyses.

### Histology

Testes or ovaries of adult animals were dissected in PBS solution and placed in 10% formalin. Tissue sections and hematoxylin/eosin staining was performed by American Histo Labs Inc.

### Quantitative PCR

Total RNA was extracted using RNeasy plus mini kit (Qiagen) and the cDNA was prepared using the Trancriptor First Strand cDNA Synthesis Kit (Roche). Quantitative PCR was performed using a Maxima SYBR Green/Rox Kit (Fermentas) according to the manufacturer’s instructions for a 7500 Real-Time PCR system. Gene copy number was calculated with ABI SDS Software. PCR amplification of the coding region of β actin gene was used for normalization. The following primer pairs have been used: *Spo11 beta*: CTCTAGTTCTGAGGTTCTTACAGCT, GGACAATACTTTCAGAATCAGAGCG; *Spo11 alpha*: GCGTGGCCTCTAGGTTTGATGATT, TCATCGATGGCGCTGTCCAC; *ActB*: CCAACTGGGACGACATGGAG, CCAACTGGGACGACATGGAG.

### Antibodies

Rabbit anti-DMC1 antibodies to a full-length protein were generated by New England Peptide and affinity purified. Mouse anti-SCP3 antibodies were previously described [34]. Rabbit anti-SCP1 and anti-SCP3 antibodies were a gift from C. Hoog. The following commercial antibodies have been used: Anti-H3K4me3, Milipore (07–473); anti-γH2AX, Trevigen (4411-PC-100); mouse anti-MLH1, BD Biosciences (551092); rabbit anti-RAD51 and goat anti-DMC1, Santa Cruz (H-92 sc-8349 and C-20 sc-8973, respectively). Secondary antibodies were from Jackson IR Laboratories.

### Meiotic chromosome spreads

Spermatocytes: Seminiferous tubules were chopped in RPMI 1640 high-glucose media (GIBCO-BRL). The cells were released from the tubules by pipetting and filtered through a 40 μm cell strainer (Falcon). The cells were pelleted and washed with RPMI. The resulting pellet was resuspended in 0.5% NaCl, added to the glass slides, and allowed to adhere for 10–15 min. The slides were fixed in 2% paraformaldehyde with 0.03% SDS for 3 min, 2% paraformaldehyde for 3 min, washed 3 times in 0.4% Photo-Flo 200 (Kodak) for 1 min, and air dried.

Oocytes: Ovaries were dissected in PBS at E15.5 for DMC1 staining and at E18.5 for MLH1 staining. Ovaries were placed in 20 μl of 100 mM sucrose, disrupted with tweezers and pipetted up and down until cell suspension was formed. The cells were added to 100 μl of 1% paraformaldehyde, 0.1% Triton X-100 solution that was spread over the slide. Slides were kept 2-4 h in humidified chamber at room temperature, then air dried. After four 1 min washes in 0.4% Kodak Photo Flo the slides were air dried again.

### Immunofluorescence

The slides were incubated with blocking solution (1% donkey serum, 0.3% BSA, 0.005% Triton X-100 in PBS) for 20 minutes at 37°C in a humidity chamber. Primary antibodies were diluted in blocking buffer and incubated under the same conditions for 1–2 hr. After two 5 min washes in 0.4% Photo-Flo/PBS solution, slides were blocked for an additional 5 min and incubated with secondary antibodies for 20 min at room temperature. The slides were washed twice with 0.4% Photo-Flo in PBS, rinsed twice with 0.4% Photo-Flo, and allowed to air dry.

### Chromatin immunoprecipitation and high throughput sequencing

Chromatin immunoprecipitation was done as previously described [19,23] with small modifications. Testes were fixed for 10 min in 1% formaldehyde. After quenching the tissue was homogenised, filtered through 40 μm cell strainer, and washed in the following buffers: 1) PBS (twice); 2) 0.25% Triton X-100, 10 mM EDTA, 0.5 mM EGTA, 10 mM Tris pH8; 3) 0.2 M NaCl, 1 mM EDTA, 0.5 mM EGTA, 10 mM Tris pH8. Cells were lysed in 1.5 ml of the lysis buffer (1% SDS, 10 mM EDTA, 50 mM TrisCl pH8 with complete protein inhibitor cocktail (Roche) and the chromatin was sheared to ~1000 bp by sonication. The sample was dialyzed against ChIP buffer (0.01% SDS, 1.1% Triton X-100, 1.2 mM EDTA, 16.7 mM TrisHCl, 167 mM NaCl). Chromatin was incubated with appropriate antibodies overnight at 4**°**C followed by 2 h incubation with Protein G beads. Beads were washed in the following buffers: 1) 0.1% SDS, 1% Triton X-100, 2 mM EDTA, 20 mM TrisHCl, 150 mM NaCl; 2) 0.1% SDS, 1% Triton X-100, 2 mM EDTA, 20 mM TrisCl pH8, 500 mM NaCl; 3) 0.25 M LiCl, 1% Igepal, 1 mM EDTA, 10 mM TrisCl, pH8, 1% Deoxycholic acid; 4) TE (twice). The chromatin was eluted by 1% SDS, 0.1 M NaHCO3 pH9 at 65°C and crosslinking was reversed at 65**°**C overnight. DNA was deproteinized for 2 h at 45**°**C and DNA was purified with a MinElute Reaction Clean up kit (QIAGEN).

Sequencing library construction was done according to the SSDS protocol that was previously described [19,50]. Sequencing was performed on an Illumina HiSeq 2000 using the standard paired-end cluster generation kit and sequencing reagents.

### Samples (Table 2)

**Table 2:** The list of samples used in this study

***Mouse strain***	***Replicate***	***Antibody***	***Method***	***Accession #***
*Spo11*^*Gal/Gal*^	1	Dmc1	SSDS	GSM1179920
*Spo11*^*Gal/Gal*^	2	Dmc1	SSDS	GSM1179921
*Spo11*^*Gal/-*^	1	Dmc1	SSDS	GSM1179919
*Spo11*^*Gal/Gal*^*Hop2*^*−/−*^	1	Dmc1	SSDS	GSM1179922
*Hop2*^*−/−*^	1	Dmc1	SSDS	GSM1179917
*Hop2*^*−/−*^	2	Dmc1	SSDS	GSM1179918
*Wild type (C57Bl/6)*	*	Dmc1	SSDS	GSM869781*, GSM869782*
*Spo11*^*Gal/Gal*^	1	H3K4me3	ChIP-Seq	GSM1179925
*Spo11*^*Gal/Gal*^*Hop2*^*−/−*^	1	H3K4me3	ChIP-Seq	GSM1179926
*Hop2*^*−/−*^	1	H3K4me3	ChIP-Seq	GSM1179924

* Published data [19].

### Hotspot identification and peak calling

We have previously shown that specific usage of ssDNA increases DSB detection sensitivity [50]. For all SSDS samples, the computational pipeline described in the aforementioned work was used to align paired-end reads to the mouse reference genome (mm9) and to identify ssDNAs. We subsequently discarded ssDNA fragments where either the first or second end read had a quality score < 30. We also retained only a maximum of 20 duplicate ssDNA fragments at any locus. Data for replicates were pooled and DSB hotspots were identified using the peak calling method described in [19]. H3K4me3 reads were aligned to the mouse mm9 genome using CASAVA 1.8. H3K4me3 peaks were called using MACS 1.3.7 and the parameters described in [19].

The effect of using different overlap windows between hotspots has been described in [19]. Overlaps between hotspots were limited to the central 400 nt. Overlaps between hotspots and H3K4me3 were limited to the central 1 Kb. Overlaps between hotspots and Gal4 binding consensus were limited to the central 2 Kb.

### Motif search

GAL4 binding sites were identified using an exhaustive genomic search for the consensus CGGN_11_CCG. No mismatches were permitted. Sites matching the putative C57Bl/6 PRDM9 binding site were identified with MAST, using standard parameters.

## Availability of supporting data

All sequencing data for this study are publicly available and have been deposited in National Center for Biotechnology Information Gene Expression Omnibus under accession number GSE48493.

## Abbreviations

DSB: Double stranded DNA breaks; CO: Crossing overs; PAR: Pseudoautosomal region.

## Competing interests

The authors declare that they have no competing interests.

## Authors’ contributions

FS and YP carried out most of the experiments, KB performed the computational analysis, US made the targeting vector. All authors participated in the design of the study and editing of the manuscript. RDCO and GVP supervised the study. All authors read and approved the final manuscript.

## Supplementary Material

Additional file 1: Figure S1 The generation of the *Gal4BD-Spo11* knock-in mouse. A. Schematic of the targeting vector. Nucleotide coordinates correspond to nucleotide #1 being located at the position -7,000 upstream of the first exon of the *Spo11* gene. B. Schematic of the Southern blot strategy. Genomic DNA was cut with either NdeI+HpaI or ApaLI and hybridized with 5’ or 3’ probes, respectively. C. Southern blots for 5’ (left) and 3’ (right) homology arms. The genotype of mice (lanes 1-3) and ES clones (lanes 4-6) is indicated. Click here for file

Additional file 2: Figure S2 The strength of *Gal4BD-Spo11* hotspots correlates with the strength of hotspots in wild type mice. The number of ssDNA fragments in wild type (C57Bl/6) hotspots was calculated for each dataset. Density scatter plots are shown for all hotspots. Log(strength) is shown on the y-axes. The Spearman Correlation Coefficient is also shown between all samples (inset). Over 94% of *Gal4BD-Spo11 Hop2-/-* hotspots corresponded to with type hotspots. Click here for file
