# Supplementary figures and images for: Suppression of genetic recombination in the pseudoautosomal region and at subtelomeres in mice with a hypomorphic Spo11 allele
Source: BMC Genomics. 2013 Jul 22;14:493. doi: 10.1186/1471-2164-14-493 (PMC3729819; doi:10.1186/1471-2164-14-493)

**A**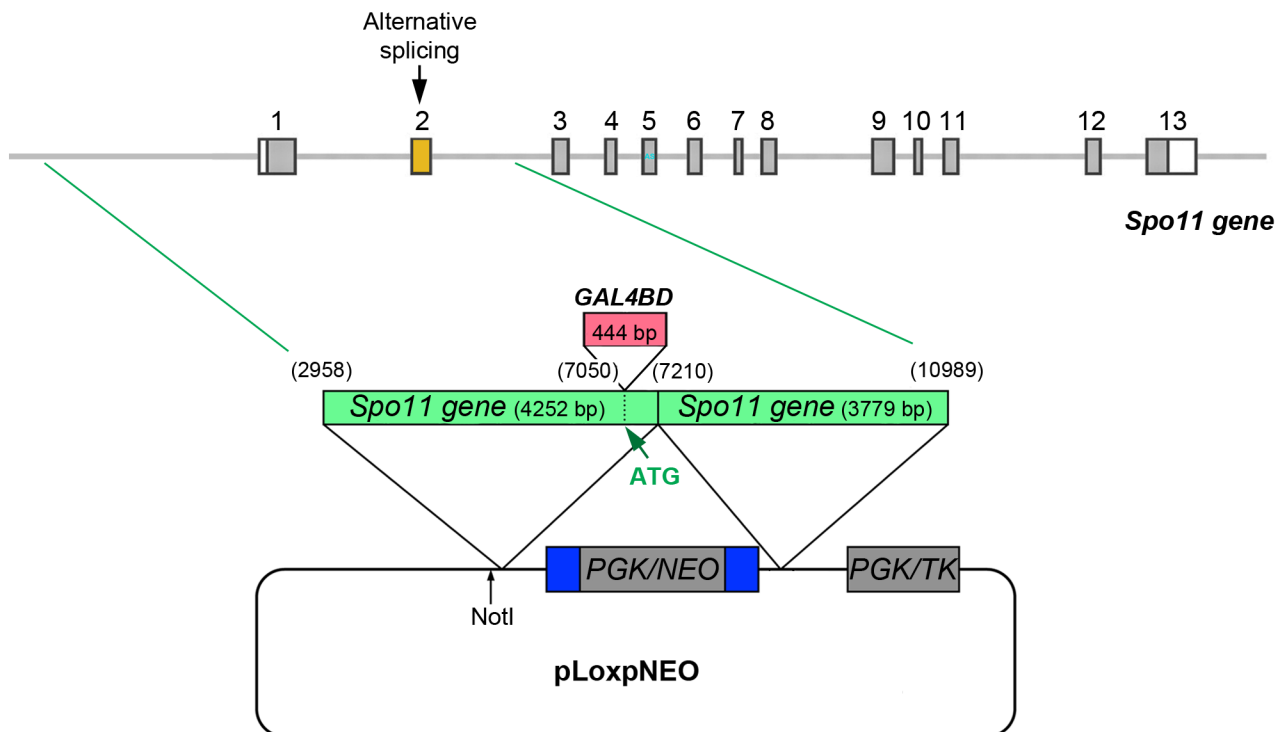**B**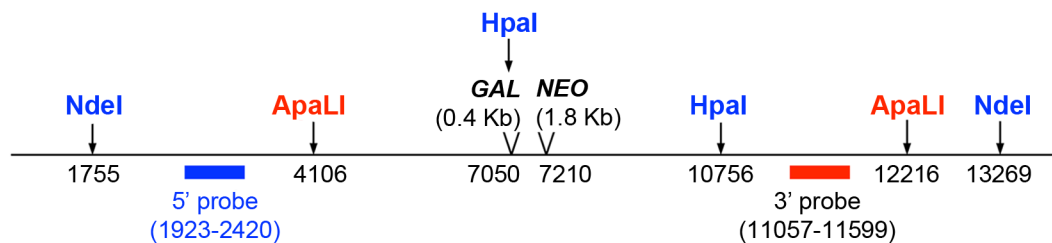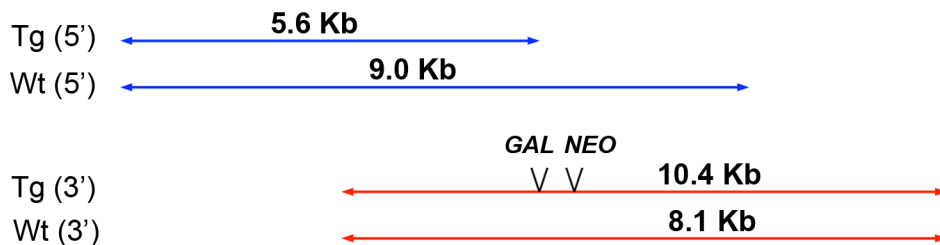**C**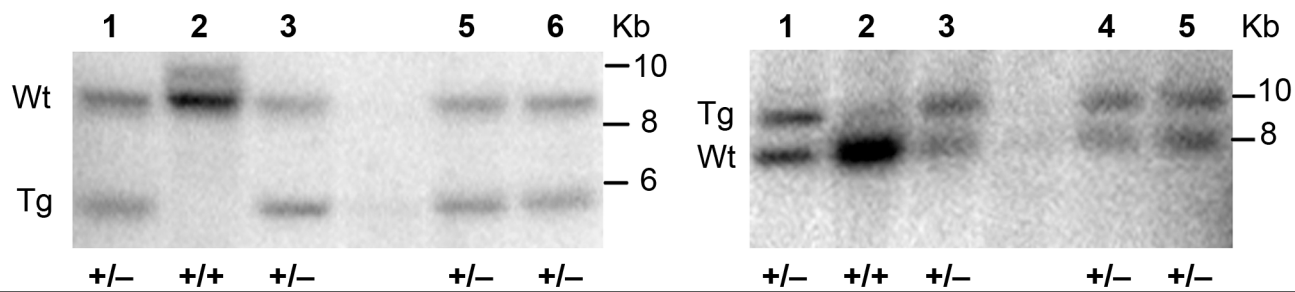

Supplement: Additional file 1: Figure S1 — The generation of the Gal4BD-Spo11 knock-in mouse. A. Schematic of the targeting vector. Nucleotide coordinates correspond to nucleotide #1 being located at the position -7,000 upstream of the first exon of the Spo11 gene. B. Schematic of the Southern blot strategy. Genomic DNA was cut with either NdeI+HpaI or ApaLI and hybridized with 5’ or 3’ probes, respectively. C. Southern blots for 5’ (left) and 3’ (right) homology arms. The genotype of mice (lanes 1-3) and ES clones (lanes 4-6) is indicated. [file 1471-2164-14-493-S1.pdf]

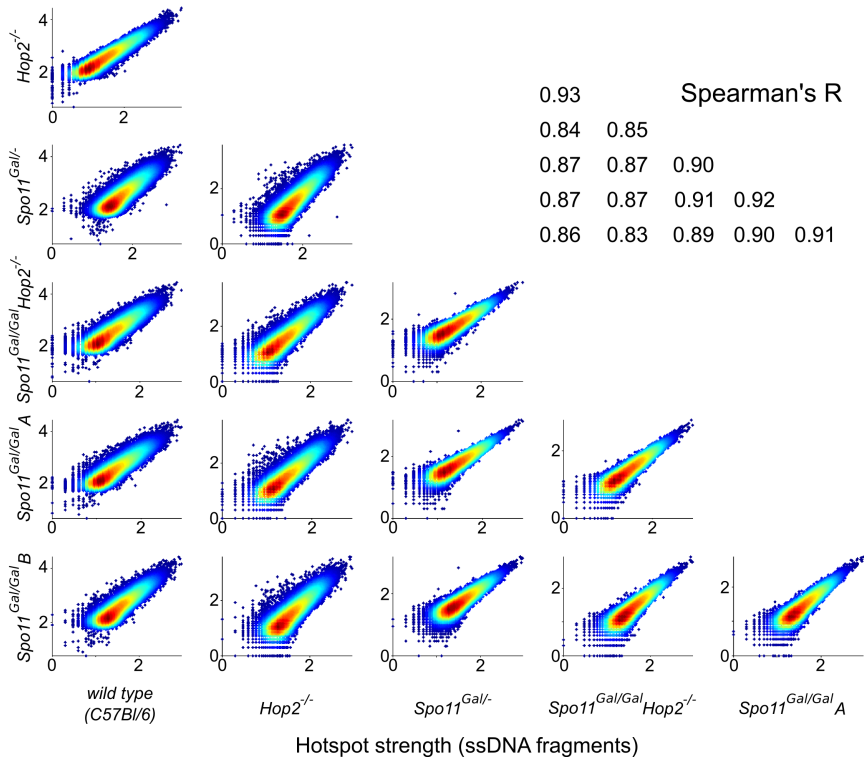

Supplement: Additional file 2: Figure S2 — The strength of Gal4BD-Spo11 hotspots correlates with the strength of hotspots in wild type mice. The number of ssDNA fragments in wild type (C57Bl/6) hotspots was calculated for each dataset. Density scatter plots are shown for all hotspots. Log(strength) is shown on the y-axes. The Spearman Correlation Coefficient is also shown between all samples (inset). Over 94% of Gal4BD-Spo11 Hop2-/- hotspots corresponded to with type hotspots. [file 1471-2164-14-493-S2.pdf]
